# Testosterone, Cortisol and Financial Risk-Taking

**DOI:** 10.3389/fnbeh.2018.00101

**Published:** 2018-05-16

**Authors:** Joe Herbert

**Affiliations:** John van Geest Centre for Brain Repair, Department of Clinical Neurosciences, University of Cambridge, Cambridge, United Kingdom

**Keywords:** testosterone, cortisol, finance, decision-making, risk appetite, emotion, cognition, amygdala

## Abstract

Both testosterone and cortisol have major actions on financial decision-making closely related to their primary biological functions, reproductive success and response to stress, respectively. Financial risk-taking represents a particular example of strategic decisions made in the context of choice under conditions of uncertainty. Such decisions have multiple components, and this article considers how much we know of how either hormone affects risk-appetite, reward value, information processing and estimation of the costs and benefits of potential success or failure, both personal and social. It also considers how far we can map these actions on neural mechanisms underlying risk appetite and decision-making, with particular reference to areas of the brain concerned in either cognitive or emotional functions.

## Introduction

Many hormones may be able to influence financial decision-making, but two stand out as prime candidates because of their biological functions. Testosterone has well-established roles in reproduction, which embrace aggression, competitiveness and risk-taking, all essential elements of financial dealings as well as successful reproduction. Professional finance is primarily the province of males, though the situation is slowly changing; the financial world has been largely constructed by males and this reflects how hormones influence it. Cortisol is a fundamental component of the response to stress and is important for coping with unpredictable or threatening events, also a common feature or consequence of financial decisions, particularly those made under conditions of duress. Although the role of each hormone is usually considered separately, it must be recognized that under real-life conditions both will be operating together in the same individual. Because hormonal events are not apparent to the individual concerned, their influence on decision-making is covert. Furthermore, levels of hormones, the way they respond to events, and the effects these changes may have on the brain and behavior are all individually variable. So, although it is possible to define an overall action of both testosterone and cortisol on financial behavior in general, and risk-taking in particular, it is equally important to take into account those other factors, genetic or experiential, that modify endocrine responses and the effects they have in individual cases. Most of these have yet to be studied.

## What Is Risk?

Risk appetite is the propensity to take risks: risk-seeking is the behavior that may, or may not, follow a given level of risk appetite. Risk occurs when there is more than one outcome when pursuing a desirable goal, in which one or more of these outcomes may be lower than the safe alternative and thus result in relative or absolute loss, danger or other undesirable consequences. In the more restricted context of finance, risk as outcome variance contributes to the subjective value an individual attaches to that risky option. The subjective value derived from risk is typically determined by giving individuals a choice between a safe (i.e., risk-free) and a risky alternative. If one adjusts the magnitude of the safe alternative until the decision maker is indifferent between the two alternatives, one has determined the subjective value of the risk. Individuals who are risk averse give up money to avoid risk. That is, they are indifferent at safe magnitudes that are smaller than the expected value of the risky alternative. Conversely, individuals who are risk-seeking pay money in order to experience risk. The important point here is that it is the subjective, not the objective, value of the reward and the perceived (rather than the actual) probability of success that influences risk-taking.

Risky decision-making involves several distinct components. Information about the likelihood of success of a particular action is the first, and this depends on previous experience of similar situations, the amount and accuracy of current information, and the ability of the individual to assess that information. From this information, the risk-taker estimates the probability of success and the consequences of failure. The decision to take a given action depends on the subjective value of success or failure to the individual concerned (utility), which can include personal consequences directly related to the decision (e.g., immediate loss or gain of money) or secondary ones (social esteem, promotion, loss of job or livelihood). Major theoretical accounts of risk valuation include expected utility theory, prospect theory and the summary statistics approach to finance theory (reviewed in Schultz, 2006; D’Acremont and Bossaerts, 2008). One problem with many theories of economic risk-taking is that they attempt to cover all contexts and eventualities. But there are substantial differences between, say, a professional trader with much experience and specific training, dealing in millions of pounds every day upon which his salary and even his employment depends, and an average citizen, untrained and inexperienced in financial matters, making everyday financial decisions, some of which may have little consequence. Attempts to devise a more comprehensive theoretical base for economics continue (Orrell, 2018).

There are different types of risk, including liquidity risks, sovereign risks, insurance risks, business risks, default risks etc. Mathematical definitions of risk mostly assume that rewards fluctuate around the mean value (variance) but other patterns include situations in which high reward occurs only occasionally (positive skewness) or scanty reward occurs often (negative skewness; Genest et al., 2016). Most of the literature on the role of hormones in finance focuses on rapid decisions made under the artificial conditions of the laboratory that attempt to reproduce, to some extent, those made in real life within a narrow definition of risk (see below).

Financial decisions and assessments of associated risks are in many ways no different from other types of decisions (Kusev et al., 2017). In particular, decisions taken in contexts of violence or combat have many of the same properties (see below). Both may require rapid decisions, based on estimates of current information which may be available in rapidly changing amounts and to varying degrees of accuracy. Much of the literature on risk-taking in other contexts, particularly those that include urgent and personally-important outcomes, will therefore be highly applicable to understanding the basis of financial risk-taking, even if they have not been directly tested. It should be noted that these circumstances, historically at least, have been mostly masculine ones, a point considered further below. The major difference is that financial risks involve the loss or gain of money rather than personal danger or physical assets. But money represents both potential gain of assets and alterations in social and personal status, factors which are not so different from the more traditional objectives of personal conflict or war or assets such as territory, food supply or sexual partners (Slovic, 1964). A major difference between money and these more biological rewards (based on current or anticipated need) is that gain or loss of money does not necessarily apply to any particular primary reward, such as food, drink or sex. Furthermore, unlike these primary rewards, the rewarding nature of money has to be learnt, and varies with culture and circumstance.

Both testosterone and cortisol have central roles in these behaviors. In both situations, not only the outcome but also the actions associated with risk-taking may themselves be important, since display of such behaviors may have social implications for esteem or leadership, and may therefore contribute to the decision-making process (Eckel and Grossman, 2002). It follows that the neural and endocrine mechanisms associated with neuroeconomics will resemble those in other behavioral contexts involving evaluating risks and making decisions, and the extensive psychological literature on learning and reward assessment will also have direct relevance (Camerer, 2008).

The notion that financial decisions are always taken as a result of accurate and objective assessments of risks and benefits has long since been superseded by a more nuanced approach; in particular, psychological theory realized that risk needs to be perceived and that emotional factors as well as cognitive processes can influence this perception and the decisions that follow from it (Kahneman and Tversky, 1979). Distinctions between “emotion” and “cognition” are difficult and not always clear, and the contribution of either depend not only on the current assessment of a risky choice but on such general properties as personality, emotionality and current mood as well as experience, training, and the particular properties and circumstances of the choice to be made and how they are computed (Zuckerman, 1991). We shall need to consider which components of this manifold system are controlled or influenced by hormones. There is an extensive account of the theoretical basis of risk and decisions made under conditions of uncertainty (Starcke and Brand, 2012).

This article focusses on the roles of testosterone and cortisol in acute decisions made under such uncertainty. As outlined above, such decisions are common in finance, but also in other aspects of life. We can therefore apply some of the information on the way these two hormones affect behavior to the more particular context of finance. The choice of these two hormones rests on the knowledge that they are the ones most obviously concerned with some of the fundamental aspects of behavior that occur under conditions when rewards are only obtainable if there is an assessment of the associated risks, culminating in decisions about whether or not to take them.

## Similarities and Differences Between Testosterone and Cortisol

Though both testosterone and cortisol have powerful influences on decision-making, there are important differences as well as similarities between the hormones themselves. Both are steroids, which means that the cellular action they have on neurons is similar to the extent that both act on intracellular steroid-binding molecules, receptors, which are reasonably but not entirely specific for each hormone (Claessens et al., 2017; Gray et al., 2017; Maney, 2017). There is also evidence for a second, more rapidly acting membrane-bound receptor for both steroids (Vernocchi et al., 2013; Shihan et al., 2014). Thus the neural actions of both hormones can be both rapid (within a few minutes) via the membrane-located receptors or more prolonged (hours or days), since the intracellular receptors, when activated by a bound steroid, act directly on the genome though on different elements—either glucocorticoid or androgen receptor binding sites. In each case, there are large numbers of downstream genes that are either activated or suppressed as the result of this addressing of the genome. The respective patterns of this genomic response, and how they differ between the two steroids, have not been adequately elucidated.

Access to the brain is essential if they are to influence behavior and this is regulated in a similar way for both steroids. Secreted testosterone and cortisol bind to large plasma proteins, either sex-hormone or corticoid binding globulin (SHBG, CBG). These carrier proteins limit access to the brain because only unbound (“free”) steroid can pass through the blood-brain barrier. So alterations on either the proportion of steroid binding to its respective globulin, or the levels of that globulin, will influence how much reaches the brain irrespective of blood levels. However, as blood levels rise there will come a point at which the carrier globulin is saturated: this will result in any extra steroid being immediately available for entry to the brain, and therefore a disproportionate surge of intracerebral hormone. This may have important consequences for behavior.

There are also significant differences between testosterone and cortisol. Both steroids are secreted in a series of c. 90 min (circhoral) pulses. Testosterone levels have a minor daily rhythm whose physiological significance has never been shown. Cortisol has a major rhythm, with morning levels being 4–5 higher than those in the evening (the amplitude is individually very variable; Bailey and Silver, 2014). Both the circhoral and daily rhythms of cortisol have coding properties for the expression of corticoid-sensitive genes (Russell et al., 2015; Lightman, 2016; George et al., 2017). Disturbances in the daily rhythm (e.g., during episodes of stress or depressed mood) will alter this coding property, but this can be distinct from increases in overall exposure of the brain to cortisol. Both may have neurobiological consequences (Herbert et al., 2006). There are marked gender differences in testosterone levels, but much less in cortisol, though morning cortisol levels are around 20% higher in females (Netherton et al., 2004). Adult male testosterone levels are very labile and environmental events that are very relevant to financial decisions, such as a psychological or physical challenge or success in a competitive encounter, raise levels whereas situations of persistent stress or fear lower them (Archer, 2006; Goetz et al., 2014). There is also a gradual decline with age, though this is also individually variable (O’Connor et al., 2011). Cortisol rapidly responds to stressful events, particularly those that are threatening, unpredictable and lack evident means for coping with them, including social or material support (Lucassen et al., 2014). This stress-related increase is an essential part of the response to adversity. Unlike testosterone, very low levels or absent cortisol (Addison’s disease) are life-threatening.

There are well-known genetic variations in the androgen receptor which have significant consequences for its function. The length of the CAG repeat at the N-terminal has a reciprocal effect on testosterone sensitivity, and is individually variable (Morimoto et al., 1996). This will moderate the behavioral effects of testosterone in an individual manner, but has seldom been taken into account. Other, less common, variants include some that prevent testosterone from acting on the brain, resulting in a female phenotype in an XY individual (Wisniewski et al., 2000; Jääskeläinen, 2012). Genetic variants in the glucocorticoid receptor are also known; there is no coherent account of their physiological significance, though they have been implicated in vulnerability for depression (Wüst et al., 2004; van Rossum et al., 2005; Bustamante et al., 2016). Some of the behavioral effects of testosterone depend on aromatization to estrogen (Finkelstein et al., 2013). Genetic and other moderators of aromatization will therefore affect the behavioral consequences of altered testosterone. The actions of cortisol on the brain do not depend on an equivalent mechanism, but conversion to inactive cortisone by 11β-hydroxysteroid dehydrogenase (also genetically variable) protects mineralocorticoid receptors from its action (MacLullich et al., 2012). Altered conversion of cortisol to other metabolites in the brain is another individual difference (Alikhani-Koupaei et al., 2007; Ragnarsson et al., 2014). The areas of the brain on which the two steroids act are also different and are discussed in more detail below. There are also proposed interactions between testosterone and cortisol—the behavioral effects of changes in one depending on levels of the other (Mehta and Josephs, 2010) that will also be considered further below.

## Lifetime Trajectories in Testosterone and Cortisol

The lifetime trajectories of the two steroids differ. The human male brain is exposed to three successive waves of testosterone (Nieschlag and Behre, 2012). The first, beginning at around 10 weeks post-fertilization, has major effects on the organization of the brain, particularly sexual identity, preference and behavior and sensitivity to testosterone in adulthood, though other aspects of testosterone-related functions may also be affected. The second surge lasts around 4 months postnatally, and lasts about 16 weeks. Its function is still largely mysterious. The third surge is responsible for puberty and its associated physical and psychological events, and lasts for the remainder of the male’s life, though levels may decline with age (Lewis et al., 1976). Cortisol does not show these age-related surges, though adverse events early in life may alter subsequent levels or the way they respond to stress: labeled “re-programming” (Pearson et al., 2015; see below) and levels may increase with age (Wrosch et al., 2007; Lupien et al., 2009). Moreover, there may be significant sex differences in the way that cortisol affects decision-making (van den Bos et al., 2009) since males make most of the financial decisions under the conditions considered here, this will be our focus. However, changes in the financial industry in the future may alter this perspective.

## Assessing the Roles of Hormones in Risk-Taking

There are several methods of assessing the roles of hormones in financial decision-making, none of them entirely satisfactory (this also applies to other studies of risk appetite). The first, essentially correlational, is to relate differences in levels of testosterone or cortisol, or changes in those levels, with liability to take risks or avoid losses. The advantages of this method are that it allows observations to be made under real-life conditions: the disadvantage is that can never establish causality. The most direct method is to give steroids (e.g., testosterone or cortisol) to those engaged in finance (e.g., daily trading) and measure the outcome. This is legally, practically and ethically impossible, as is giving androgenic steroids to competitive athletes. But steroids can be administered to subjects under experimental or laboratory conditions, in which they play games that are designed to reproduce at least some of the features of real life. However, it should not be forgotten that these experimental conditions never reproduce, entirely, the conditions and consequences of real-life financial dealings.

Levels of testosterone are only one way of assessing changes in its activity: the effect it has on behavior will vary according, for example, to genetic variance in the androgen receptor or SHBG, and the pattern of other genes with which testosterone interacts, as well as factors such as the “personality” and experience of the individual concerned. Similar reservations apply to cortisol. So far, there are no studies on the genetic make-up of professional financiers (e.g., traders) or those making everyday financial decisions, and how it might be related to performance under various conditions and relate to changes in hormone levels. Similar considerations apply to investigations in which subjects play a financial game which has some similarity to real-life conditions (though usually less complex and demanding; Cueva et al., 2015; Schipper, 2014). Under these conditions it is possible to give hormones (e.g., testosterone or cortisol), though the fact that the rewards or the consequences are seldom very significant for the subjects robs such studies of an important real-life element. Since risks are a component of other activities, it should also be possible to extrapolate from non-financial studies to yield a greater understanding of financial risk-taking, after taking any special features into account. Experimental studies on risk-taking and reward-related behavior in animals are collateral evidence, though the differential cognitive abilities of human and animal brains limit their usefulness. Nevertheless, the basic neural mechanisms may be similar, and there are greater opportunities for experimental manipulations and examination.

## Testosterone and Adolescent Risk-Taking

The surge in testosterone that occurs at puberty and during adolescence is associated with increased appetite for risks and rewards including those related to financial gain in both sexes, particularly as these affect peer relationships and social status, perhaps most prominently in boys (Morrongiello and Rennie, 1998; Steinberg, 2008; Vermeersch et al., 2008; Cardoos et al., 2017). There is increased activation of the nucleus accumbens, an area associated with reward (see below) though this was not related to individual testosterone levels (Alarcón et al., 2017). The neuroendocrine explanation for this has focused on the role of dopamine, referring to its well-known role in the neural basis of reward (Schultz, 2006). There is experimental evidence that dopamine is necessary for testosterone-induced motivated behavior, and that testosterone also moderates dopamine transporters and receptors in the substantia nigra (Bell and Sisk, 2013; Purves-Tyson et al., 2014; Morris et al., 2015).

However, in humans there is an additional factor: the maturation of the frontal lobes. Progressive reduction in the age of puberty has resulted in a mismatch between the advent of the pubertal testosterone surge and the maturation of the brain, particularly the frontal lobes (late adolescence, early 20 s). Furthermore, the frontal lobes mature later in boys than girls (Lenroot and Giedd, 2010; Raznahan et al., 2010; Mills et al., 2014). Since this part of the brain plays an established role in the evaluation of rewards and associated risks (see below), as well as in the emotional response to them, the increasing mismatch between the endocrine and neural events now occurring at puberty may well play a crucial role in adolescent risk-taking, including those associated with financial decisions. For example, pubertal testosterone increases the responses of the frontal lobe to emotional events (Tyborowska et al., 2016). It is interesting to speculate whether increases in the utility of financial gains at puberty might be secondary to the advent of sexual motivation. Testosterone (in both sexes) heightens sexual motivation (reward). This increases the utility of money, in the sense that it may promote access to sexual objectives either directly or by increasing social status. It may be one example of how hormones, through their selective action on reward value, can alter the pattern of financial risk-taking in a setting that ostensibly has no relation to the primary action of that hormone, in this case testosterone on sexual motivation. It should also be noted that the pubertal surge of testosterone in males, unlike females, acts on a brain that has already been exposed to the same hormone prenatally, an event which may sensitize it to the pubertal surge as well as influencing the nature of the behavioral response to it (Apicella et al., 2008).

## Testosterone and Risk-Taking in Adults

The impact of the basic reproductive function of testosterone and its influence on financial risk-taking is supported by other experiments on adult men. Heterosexual men exposed to opposite-sex stimuli take greater financial risks (Baker and Maner, 2008). This suggests sexual motivation, which is testosterone-dependent, accentuates risk-taking as part of the process of getting a mate (display, increased assets, etc.). However, images of physically-attractive men also increase risk-taking (also in heterosexual subjects), suggesting that this stimulus acts on the competitive element of sexual selection (Chan, 2015). As part of its widespread effects on behavior, all related to its fundamental role in reproduction, testosterone helps to maintain social status, and levels can reflect social or physical challenge as well as status (Booth et al., 1989; Mazur and Booth, 1998). This may influence risk-taking as part of the competition to sustain that status (Stanton and Schultheiss, 2009). However, there may also be a reciprocal interaction between social status and testosterone: men with lower testosterone put into a high status position showed poorer cognitive functioning that those with higher testosterone: the reverse occurred after being put into lower status positions (Josephs et al., 2006). There seems to be a variety of ways, all related to sex or its concomitants, but differing in proximal mechanisms, by which testosterone-related behavior could alter financial risk-taking.

Studies on the association between testosterone levels and financial trading in real-life contexts and have provided intriguing findings—traders made more money on days when their testosterone levels were highest (Coates and Herbert, 2008). This agrees with laboratory studies showing that subjects with higher testosterone levels made riskier bids in a financial game (Apicella et al., 2008), though this has not always been confirmed (Sapienza et al., 2009). These findings are associations, and unless there is considerably more information on individual strategies, supported by interventional studies, the level of analysis is limited. Interestingly, giving testosterone to traders playing an economic game that resembled real-life resulted in increased price offers (i.e., mispricing) and over-optimism about future changes in asset values (Nadler et al., 2017) and non-professional subjects showed similar effects, together with increased appetite for risk (Cueva et al., 2015). Thus, testosterone appears to increase individual willingness to take financial risks because it biases estimates of outcome. It is interesting to speculate that collective over-ambitious estimates may be one reason for the periodic “bubbles” that affect the stability of financial markets (see below). Whether the “winner” effect—increased levels of testosterone after a successful deal—has any effect on subsequent risk-taking has not been established, though it remains a possibility. Men playing with a gun (but not a children’s toy) showed increased testosterone, and were more willing to inflict discomfort to others (adding a hot sauce to food; Klinesmith et al., 2006), suggesting that similar “carry-over” effects may occur in a financial setting, and there are associations between acute changes in testosterone following a competitive challenge and features such as subsequent competitiveness, aggression and rating faces as trustworthy (reviewed by Apicella et al., 2015) though whether these depended upon increased testosterone or on related psychological traits independent of the actual rise in testosterone remains speculative. A recent history of receiving rewards can reset estimation of future rewards (Khaw et al., 2017), though whether the response of either testosterone or cortisol to such previous rewards contributes to this effect is not yet known. Note that there have been no substantive assessments of the role of other testosterone-related features, including genetic variants of the androgen receptor, in financial risk-taking behavior.

We should not be surprised if testosterone has manifold actions on financial decision-making. A similarly wide canvas is seen in its primary role in reproduction. In order to achieve its role, testosterone has to act on both physical features, such as the growth of horns, teeth and muscles, as well as on a range of behavioral attributes such as aggressiveness, competitiveness and willingness to take risks, in addition to primary actions on sexual motivation and attractiveness (Herbert, 2017).

## Gender Differences in Risk Appetite

Gender differences in risk appetite are an indirect and incomplete way of assessing the effects of hormones, particularly testosterone. It is important to recognize that not all gender differences are testosterone-based. The Y chromosome expresses genes that directly affect behavior, and the presence of two X chromosomes in females is also important. But more significantly, environmental factors such as upbringing, expectations, opportunities and social attitudes, though directly related to gender, are an indirect effect of testosterone-dependent gender differences in the brain and its phenotype and can have potent actions on any aspect of gender-related behavior, including the perception of risk and risk-taking (Lenroot and Giedd, 2010). Nevertheless, careful assessment of gender differences in risk appetite or processing can add some information about testosterone-dependent aspects of risk-taking, though it may be difficult to separate the role of early exposure to testosterone from the action of post-pubertal hormone (see below), and to account for the effects of social attitudes and expectations.

First, the nature of the risk is important. Many studies show that males and females differ with respect to the kinds of risks they find attractive or aversive (Schubert et al., 1999; Rolison et al., 2014). A meta-analysis of risk-taking across several domains showed that males were generally more inclined to take risks than females, though the size of the effect varied with different risks. Gambling, for example, showed a greater gender difference than risky sexual behavior, but less than physical risk-taking (Bryrnes et al., 1999). More recent work has moderated this view: risky social behavior either shows no gender difference or more risks were taken by females; the greater appetite for financial risks (e.g., gambling) by males was confirmed, women being more pessimistic about a positive outcome and enjoying it less (i.e., reward value; Harris and Jenkins, 2006).

Giving testosterone to women and then assessing the effect it has on risk-taking has dubious value if it is regarded as a test of gender differences (i.e., making women more “male-like”) or a demonstration of the action of testosterone in general, since this ignores both the gender difference in early exposure to testosterone, and the presence or absence of two X or one Y chromosomes. Bearing this in mind, exogenous testosterone increases stress reactivity in women (startle reflex; Hermans et al., 2007) and decreases empathy (which is generally greater in women than men; Hermans et al., 2006). This will impact financial decisions, since stress and empathy both affect risk appetite and concepts of fairness and are examples of the interaction between stress (cortisol) and testosterone (see below). Gender differences in risk-taking have been related to corresponding differences in the 2D:4D digit ratio, proposed to be a reliable index of exposure to early testosterone in females as well as males (van Honk et al., 2011); however, there are serious questions about the information given by the digit ratio.

Since prenatal testosterone has such a powerful effect on subsequent behavior and physiology in males, there is considerable interest in estimating its action in individual cases. It should be noted that this depends not only on levels of testosterone, but also on the sensitivity of response to it, which includes genetic variation in the androgen receptor (Vermeersch et al., 2010; Hurd et al., 2011). Direct measurement of testosterone during the critical period (c.10–20 weeks) is not possible. The 2D:4D digit ratio has been used as a proxy, but this is highly dubious. The ratio is less in males than females (though there is a considerable overlap; Manning et al., 1998; Breedlove, 2010; Knickmeyer et al., 2011); XY individuals with complete androgen insensitivity have ratios in the female range (van Hemmen et al., 2017). Prenatal testosterone thus plays a role in determining the ratio (which has no known function), but this is very different from concluding that individual differences in prenatal testosterone are reflected in individual measures of the digit ratio in males, for which there is no convincing evidence (see Ventura et al., 2013). Yet the ratio, which is easily measured, continues to be used in this way (e.g., Kim et al., 2014). Lower ratios in males have been associated with higher risk taking (the opposite was found for females), though this was attributed to greater ability for abstract reasoning as well as greater risk appetite, but only in males (Brañas-Garza and Rustichini, 2011; Branas-Garza et al., 2018). There have been both negative reports and positive ones for the association of lower digit ratios with increased risk-taking within both males and females (Branas-Garza et al., 2018) as well as with greater reflective consideration of decisions in both sexes (Bosch-Domènech et al., 2014). Lower ratios have been associated with less over-confidence in males (estimate of success in a quiz) but only when success was rewarded, suggesting that this might be related to adult surges of testosterone responding to challenge and acting on a brain pre-conditioned by pre-natal testosterone—though this was not measured (Neyse et al., 2016). This seems incompatible with a report that administration of testosterone to adult males increases optimism (confidence) about outcomes (Cueva et al., 2015). Since females are not exposed to early testicular testosterone, the rationale for relating their individual digit ratios to risk-taking seems obscure. Furthermore, the variance in digit ratios for females is quite similar to males: this suggests that factors other than prenatal testosterone influences individual digit ratios; the same may apply to males. The current uncertainty about the accuracy or validity of the digit ratio as a marker of the amount of early exposure to testosterone in individual males makes interpretation of these results both difficult and tentative.

Empathy plays a role in many financial dealings, for example in the ultimatum game, and is generally greater in women than men (Auyeung et al., 2009). Generosity in this game is reduced by giving men or women testosterone (Zak et al., 2009; van Honk et al., 2011), and men with higher levels are more likely to reject low offers (Burnham, 2007). Higher testosterone is associated with less empathy and greater “utilitarianism” in decisions that require a choice that has immediate costly consequences: this would impact financial as well as other types of decisions (Carney and Mason, 2010). It should be noted that in this context, as in all others, the actions of testosterone are only one factor determining such behavior (Takahashi et al., 2012). Entrepreneurship is a form of risk-taking and challenge, in that the participant risks assets in setting up and developing his/her own business. Whether this can be related to testosterone is disputed: males setting up a new venture had higher testosterone levels, whereas those who had ever been self-employed (a different definition) did not (White et al., 2006; van der Loos et al., 2013).

## The Complexities of Stress

Stress is often used as if it is a single defined concept. This is not the case. Stress is actually a generic term for a range of situations: the only commonality is that they represent an unusual demand which, if this is to be met satisfactorily, requires an adaptive response. But an inadequate or mal-adaptive response may also occur, with corresponding consequences. There is also confusion between stressors (the nature of the demand) and the reaction to the demand (the stress response). The response to stress (often abbreviated to “stress”) is also complex. The physiological response to an acute stress involves both catecholamines as well as cortisol, and there is experimental evidence that they interact in the brain (Ferry et al., 1999; McReynolds et al., 2010; Wolf et al., 2016). This will differentiate the effects of acute from chronic stress, since catecholamines play a lesser role in the latter. There is a recent report that increased loss aversion after cortisol administration only occurred when combined with simultaneous noradrenergic activation (Margittai et al., 2018).

Most laboratory studies of the effects of stress on decision-making focus on acute stress (Starcke and Brand, 2012) at a single time point, but another complication is that the effects of stress may alter with time. For example, an initial response may be to increase risk-appetite, but this may reverse at later time periods (Bendahan et al., 2017) either because of the altered interaction between catecholamines and cortisol, or because its initial membrane-dependent actions differ from the slower genomic ones. The nature of the stressor is also important: physical stressors, such as cold immersion (pressor test) are not the same either physiologically, cognitively or emotionally as psychological stressors such as the Trier test or cognitive overloading, such as simultaneous distractors (e.g., mathematical problems), and none of these capture all the features of the stress associated with incipient or current risky financial decisions. The latter incorporate emotional and cognitive reactions to the nature of the decision itself, which are not present in background stressors, unrelated to the risk. This may well have different consequences for decision-related behavior than other types of stress. A recent report describes distinct metabolic patterns in the hippocampus following either physical or psychological stress, emphasizing the difference between them (Liu et al., 2018). Yet all are often included in the single sobriquet of “stress” and interpreted as such. Stress is also more than elevated cortisol, though this is an important component of the stress response. A major reason for the inconsistency of reports on the effects of stress on decision-making is one result of insufficient attention to these important distinctions and variables. There is also, as already mentioned, the problem of modeling real-life situations in the laboratory.

## Cortisol and Risk-Taking

It is important to recognize the different effects of raising cortisol levels and altering the shape of the daily rhythm. Both have consequences for brain function, but they can differ (see above). Experiments that give subjects cortisol several times a day will confuse the two mechanisms (e.g., Kandasamy et al., 2014). Even though persistent stress can result in both increased cortisol and altered daily rhythms, it is important to bear this distinction in mind. In contrast to testosterone, dysregulated cortisol has been implicated in the increased susceptibility of the brain to damage by toxic agents, in heightened incidence of depression, and in the risk that depression poses for decision-making as well as for subsequent Alzheimer’s disease (Herbert et al., 2006; Herbert, 2013; Herbert and Lucassen, 2016).

Persistently high levels of cortisol, such as those in Cushing’s disease, impair cognitive function and also predispose to depressed mood (Starkman et al., 1981; Newcomer et al., 1999; Hook et al., 2007). The magnitude and duration of the cortisol response to stress in a financial context depends on many factors, of which uncertainty about market movements and their volatility are the most relevant to financial decisions (Coates and Herbert, 2008; Cueva et al., 2015). Most evidence has been on the effects of short-term cortisol administration, which is certainly relevant to real-life trading conditions. However, there will be circumstances in which subjects are experiencing more persistent stress, and therefore more prolonged elevations of cortisol, and this may have different results.

There are thus indications that acute, short-term increases in cortisol may have different effects from more long-term, chronic, ones (Lucassen et al., 2014). This would differentiate the influence that cortisol has on decisions in response to a short-term financial demand from its effect on those made during a more persistent state of stress. This separates acute responses (attention to threats, fear etc.) from those characteristic of more chronic states—which may relate to altered risk aversion (Putnam et al., 2007; van Ast et al., 2013). The intrinsic nature of the decision that has to be made is likely to be associated with a more acute cortisol response, whereas a pre-existing state, which may or may not be associated with the context of the financial risk to be taken, will result in a more prolonged cortisol reaction which may also influence that decision in a manner that is different from more acute or short-term cortisol responses (Porcelli et al., 2012). Acute administration of cortisol in other contexts increases the arousal response to stimuli, as well as enhancing the consolidation of memories of adverse events whilst reducing their recall (Abercrombie et al., 2003, 2005; Wirth et al., 2011; Wolf et al., 2016). There are similar indicators in the brain: the reaction of the amygdala (which contains profuse glucocorticoid receptors) to facial expression changes with time, an effect which has been related to its connections with the medial frontal cortex (Henckens et al., 2010). Stress has pervasive effects on cognitive functions highly relevant to finance, including selective attention, working memory, and cognitive control (Okon-Singer et al., 2015). Though it is usually assumed that the effects of stress are the result of altered corticoids, it should be recognized that there are other physiological and neurological consequences of stress that may contribute (Lucassen et al., 2014; see above). However, in one study stress increased risk-taking only in those in whom cortisol was elevated (Buckert et al., 2014), thus suggesting that it was cortisol that underpinned most of the effects of stress in this case.

Cortisol administration also impairs detection of errors (Hsu et al., 2003), a crucial element of rapid decisions made under duress. It increases appetite for risk (Cueva et al., 2015), though there is a contrary report, possibly as the result of a different regime of cortisol administration—repeated daily administration, which would alter both cortisol levels and its daily rhythm (Kandasamy et al., 2014). Note, too, that the effect of acute cortisol may be time-dependent (see above). A meta-analysis confirmed that stress increased appetite for rewards together with associated accentuated risk-taking: together, these resulted in overall disadvantageous outcomes (Starcke and Brand, 2016). Stress impairs executive functions such as attention and inhibition, task management and planning (Starcke et al., 2016). However, the exact consequences depend on the type of stress and the context in which it occurs (Starcke and Brand, 2016) since the behavioral action of cortisol is so widespread. Not all the effects of stress or cortisol are necessarily disadvantageous. Stress can be an enhancing experience, particularly if there are adequate resources for coping with it or if emotional states (e.g., anxiety) are consciously appraised (O’Connor et al., 2010; Akinola et al., 2016).

In contrast to testosterone, cortisol does not show a financial “winners” response (McCaul et al., 1992); another significant difference between the two hormones is that whilst both increased risky choices, only testosterone increased optimism about price changes; cortisol did not (Cueva et al., 2015). This suggests that while the effect of testosterone on risk-taking might be secondary to over-optimistic assessments of possible outcomes, cortisol had a more direct action on risk-appetite itself. This may be an adaptive (or mal-adaptive) response to financial situations that are unpredictable or apparently incontrollable, since cortisol responds so sensitively to such conditions. An uncontrolled stress response thus becomes a hindrance to the most advantageous courses of actions under these circumstances. It should be emphasized that nearly all these results have been obtained on male subjects and that there is no reason to assume that they might apply to females (Cueva et al., 2015).

## Individual Modulation of the Response to Cortisol

Although, as for testosterone, it is possible to make general statements about the effects of cortisol, either acute or chronic, or immediate or delayed, on decision-making, it is important to recognize that these effects can be moderated by individual characteristics. These include impulsivity, which tends to increase risky behavior (see below; Lempert et al., 2012) and state anxiety (Lempert et al., 2012) as well as cognitive style, such as rapid (“fast”, habitual) or slower (model-based) decision-making, and thus interactions between speed and accuracy (Kahneman, 2011) as well as other aspects of personality (Nicholson et al., 2005). For example, the accumulation of lifetime stress accentuates habitual responses to risky decisions only in those with slower cognitive styles (Friedel et al., 2017). The bases for these differences, which would likely include variation in genetic constitution and/or individual experience, has not been explored adequately. Early life stress can also have effects on decision-making in adulthood, particularly altering sensitivity to loss (Birn et al., 2017). Whilst the mechanism for such an influence is not yet known, it does recall the long-lasting epigenetic changes in the glucocorticoid receptor described in other contexts of early adversity (Mazur and Booth, 1998; Meaney et al., 2007; Herbert and Lucassen, 2016; Gray et al., 2017) which would have wide-ranging effects on the pattern of cortisol secretion.

Again, as for testosterone, cortisol can alter a number of parameters associated with financial decisions, including loss aversion, but also reward sensitivity as well as a tendency to favor short-term over longer-term gains (Canale et al., 2017). This is not surprising, given the widespread action of cortisol on the brain. However, it does mean that cortisol may have different consequences on risky behavior for those engaged in short-term decisions under duress (e.g., traders) from decisions made more deliberately for the longer term (e.g., stock investments).

## Impulsivity and Herding

The tendency to act on impulse, characterized by little reflection or consideration of possible consequences, and its influence on risky decisions, has already been mentioned. Another aspect is temporal discounting, the tendency to accept an immediate financial reward rather than a delayed, but greater, one. One measure of this is to increase the value of the delayed reward until the individual switches choices. The difference between this value and the immediate one is an index of temporal discounting, or impulsivity. This has to exclude circumstances that might make an immediate reward necessary (e.g., to settle a debt). Attention-deficient hyperactivity (ADHD) is a common developmental disorder characterized by impulsivity and is associated with greater risk-taking (Blomqvist et al., 2007).

Both cortisol and testosterone have been implicated in the control of impulsivity. Several studies show that the general trait of impulsivity—but particularly related to aggression—is associated with lower basal cortisol levels and a reduced response to stress (Blomqvist et al., 2007; Flegr et al., 2012; Lovallo, 2013; Brown et al., 2016). Lower levels of cortisol predicted temporal discounting in males, but this was opposite in women (Takahashi et al., 2010). Increased testosterone, or reduced cortisol/testosterone ratio, has been related to low impulse control (Pavlov et al., 2012), but rats treated with testosterone chose a larger, delayed reward compared to controls (Wood et al., 2013). However, higher testosterone was related to increased temporal discounting in males, though the opposite was recorded in women (Doi et al., 2015). This result in males is at odds with other reports linking higher testosterone with higher sensation-seeking, aggression and harmful risk-taking, though it has been suggested that impulsivity is actually a complex trait with different components (Reynolds et al., 2006; Bari and Robbins, 2013).

There is an extensive literature on the role of serotonin (but also dopamine) in impulsive behavior and the consequences this has for decision-making (Dalley and Roiser, 2012; Homberg, 2012; Bari and Robbins, 2013). There is an equivalent literature on the regulation of serotonin by cortisol (Chaouloff, 2000; Joels, 2011). Corticoids moderate the activity of tryptophan hydroxylase and thus the synthesis of serotonin, as well as the activity of several of its receptors (Hanley and Van de Kar, 2003; Mueller et al., 2011). There has been little study on whether genetic variants in serotonin-related genes could contribute to financial impulsivity, though low expression variants of the serotonin transporter (hSERT) or reduced cerebral concentrations of serotonin have been associated with an increased tendency for impulsive behavior in other contexts (Walderhaug et al., 2008; Pavlov et al., 2012; Cha et al., 2017).

Another example of socially-relevant behavior that influences risky economic decisions is “herding”, the tendency for individuals to follow a leader or trend without question. In situations of uncertainty, rational choices can be made following principles of statistical inference using Bayesian approaches and such explanations for herding lie in scenarios in which different individuals’ decisions are interdependent and reinforcing. However, a more complete approach takes into account a range of other factors from social psychology, neuroscience and even evolutionary biology (Baddeley, 2009). Herding is seen in many other species: deer run if one member of the group is startled without waiting to see the cause; if one bird takes off, the rest of the flock may well follow almost instantly. In these instances, herding is advantageous. There may be occasions when this is also true in financial contexts (“rational herding” (Devenow and Welch, 1996)); for example, when a small number of participants, or a prominent leader, really do have private information of value.

There are no studies on the effect of hormones on the tendency to herd in a financial context, but empirical observations suggest this is more likely to occur under conditions of stress or market uncertainty, particularly in individuals of lower cognitive ability and those susceptible to “framing” effects (Tversky and Kahneman, 1974, 1981; Kahneman and Tversky, 1979; Devenow and Welch, 1996; Baddeley, 2009; Zheng et al., 2010). These, as we have seen, are exactly the conditions that result in heightened secretion of cortisol: and the effects this might have on anxiety, risk-perception etc could easily be translated into an increased tendency for herding behavior, and hence market de-stabilization. Testosterone, it seems, might also have an action on the tendency to herd. If the digit ratio is accepted as an index of prenatal exposure (but see above) then a lower ratio (male-like) might encourage a more deliberate strategy (and less imitation), and adult levels greater abstract reasoning ability (Brañas-Garza and Rustichini, 2011; Bosch-Domènech et al., 2014). However, there is a marked tendency for the males of many species (including humans) to act collectively if their group is attacked or challenged. Thus the males of a group of monkeys will combine to repel an invasion of their territory by another group, putting aside intra-group competition or rank (Wrangham and Glowacki, 2012). This is a form of herding, though whether it is a direct consequent of the actions of testosterone remains possible but speculative. An fMRI study suggested that the amygdala, well-known as important for emotion and sensitive to both cortisol and testosterone, might be implicated in individual tendencies to herd (Baddeley et al., 2012). We should not forget that other hormones may play a role, including oxytocin, which influences “bonding” between individuals, and hence the tendency to imitate or follow an example (Panksepp, 1992; Olff et al., 2013).

**Figure 1:**
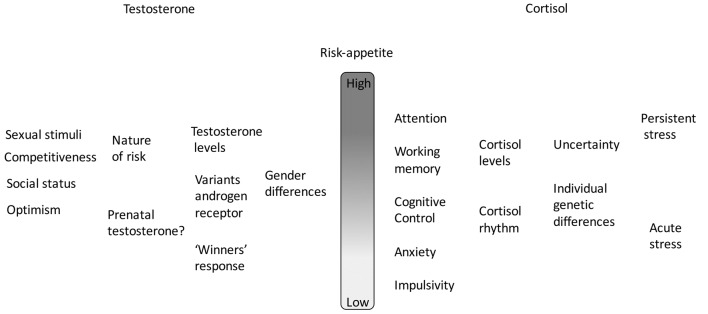
A summary of the combined actions of testosterone and cortisol on risk appetite. In each case there is a hierarchy of effects, individually variable and context-dependent.

The actions of both testosterone and cortisol on risk-appetite are summarized in Figure 1.

## Mapping the Responses in the Brain

Mapping the actions of these hormones onto the brain presents many problems. There are differences in the distribution of androgen and corticoid receptors in the brain. Androgen receptors are located mostly in limbic structures, such as the hypothalamus, amygdala and hippocampus, though there are lesser concentrations in the brainstem and deeper layers of the cerebral cortex (Simerly et al., 1990). This points to the major sites of action of testosterone on areas known to be concerned with emotion and motivation. By contrast, glucocorticoid receptors are more widely distributed, including not only limbic structures but also the cerebral and cerebellar cortices, and brain stem nuclei (e.g., those expressing serotonin or noradrenaline; Morimoto et al., 1996). This implies a different pattern of neuronal activation or inhibition which would include both emotional and cognitive functions.

It has already been pointed out that testosterone, even though its receptors are concentrated in the limbic areas, has to influence many aspects of behavior other than sexual activity in order to fulfil its primary reproductive function (e.g., aggressiveness, competitiveness, risk-taking). This variety will be reflected in the way that testosterone influences financial decisions: the effect may vary with the situation. For example, presenting sexually-related stimuli may affect decisions and their associated risks by distinct neural mechanisms, which may vary in different individuals and from situations that are more competitive or threatening (Herbert, 2017).

There is a conundrum about the role of testosterone in the brain. One way in which risk-taking varies within an individual according to context or between individuals in the same context is related to the value of the reward on offer. Most current evidence places the brain areas that respond to, anticipate, or evaluate reward in the ventral striatum, its dopaminergic innervation, or the orbital (OFC), anterior cingulate or parietal cortex (Schultz, 2004; Hsu et al., 2009; Kang et al., 2009; Kahnt et al., 2010; Louie et al., 2011; Soutschek et al., 2017). None of these forebrain areas is notable for high concentrations of androgen receptors (Rubinow and Schmidt, 1996), though they have been discerned in the midbrain dopaminergic neurons of humans and rats (Aubele and Kritzer, 2012; Morris et al., 2015). If testosterone is to bias the reward system, then there must be a link between this system and the areas of the brain (e.g., amygdala, hypothalamus, septum) responding to testosterone, and its influence on midbrain dopaminergic neurons might be one way for this to happen. There is some experimental evidence suggesting that testosterone can modulate dopaminergic activity (Purves-Tyson et al., 2014) though whether this accounts for all its actions on reward remains uncertain. This also applies to the principal action of testosterone on sexual behavior or motivation, which, as we have seen, may influence financial risk-taking. Emotion and cognition are closely interwoven, so there must be a corresponding neural representation of this association (Okon-Singer et al., 2015). Though profuse connections between, for example, the amygdala and OFC are known (Cavada et al., 2000), there is as yet no coherent account of how these bias the reward system.

The glucocorticoid receptors, having a wider distribution in the brain than androgen receptors, enable cortisol to access directly a wider neural network, hence its more general actions on cognitive and emotional functions associated with risk. But this raises questions about which particular function will predominate in a given financial situation.

A second problem is how much of the experimental work on the neural mechanisms underlying reward and choice, or the effects that stress or hormones have on these behaviors, have direct relevance to financial decisions and their associated risks humans (see above). The use of money as an asset involves cognitive and emotional processes that are not really observable in animals. Studies on the latter rely on primary rewards, such as food or palatable juice (Schultz, 2016). So much of the information on humans has to come either from studies on those with defined areas of damage to the brain, or on techniques, such as scanning, that give limited information on neural function and the way it varies both in different contexts and between individuals.

A third difficulty is that the process of risk assessment and subsequent decision-making involves a series of neural processes (as already mentioned). The perception, processing and assessment of information concerning the nature of the decision will involve several regions of the brain. Estimation of the reward value of success, or the consequences of failure involves a further process. Then comes the emotional response to the perceived risk or anticipation of success or failure. All this takes place on the background of neural states representing personality, learning, experience and knowledge of the context in which the decision is made (see above). Each stage is potentially sensitive either directly to these steroids or indirectly to their action elsewhere in the brain. Nevertheless, we would expect the actions of either testosterone or cortisol on financial decisions to reflect their primary functions: for testosterone, its central role in promoting reproductive success; for cortisol, its role in coping with stress.

The amount of information on regions of the brain involved in risk assessment and decision-making is too large to allow anything more than a summary here, with particular emphasis on whether it sheds light on the action of either testosterone or cortisol on financial risk-taking. It is generally agreed that the prefrontal cortex and its associated connections with the striatum (and its dopaminergic innervation) play a central part in recognizing risk, and deciding what action to take (Hsu et al., 2005; Holper et al., 2014; Goh et al., 2016; Ouerchefani et al., 2017). Acute stress activates a neural network that includes fronto-insular, dorsal anterior cingulate, inferio-temporal, and temporo-parietal and amygdala, thalamus, hypothalamus and midbrain (Hermans et al., 2011). The anterior insular cortex, strongly implicated in emotional expression, and with plentiful connections to the limbic brain, is also activated by risk (Mohr et al., 2010).

Risk-taking implies uncertainty about outcome. fMRI studies have suggested separate brain areas that react to uncertainty (e.g., the amygdala and orbital frontal lobe) and expected reward or its valuation (the striatum; Hsu et al., 2005, 2009; Christopoulos et al., 2009; Tobler et al., 2009; Burke and Tobler, 2011). Direct action of corticoids has been implicated in the impairment of the frontal lobes by stress (McKlveen et al., 2013, 2016); this may include alterations in dopamine release, and hence the signaling of either reward or reward errors (Butts and Phillips, 2013). Serotonin neurons also respond to reward, but differently from dopaminergic ones: dopamine may signal the relative value of a reward, whereas serotonin neurons signal its absolute value, and are inhibited by stress (Zhong et al., 2017). Though cortisol has not been directly implicated, as already mentioned there is an extensive literature on the regulation of serotonin in the brain by corticoids (Chaouloff, 2000).

Perceptual learning, and hence appraisal of risk, may also be impaired (Dinse et al., 2017). Testosterone, either acting directly or indirectly, by contrast alters the activity of the anterior insula and inferior frontal lobe (more closely associated with emotional states and the integration of risk with returns, respectively), and this is associated with increased risk taking (Tobler et al., 2009; Burke and Tobler, 2011). Both effects were moderated by genetic variants of MAOA (Wagels et al., 2017), a gene implicated in impulsivity and aggression (Dorfman et al., 2014). However, the blurred boundary between cognition and emotion is emphasized by the fact that the ventral prefrontal cortex is also concerned with reward (Juechems et al., 2017).

But the frontal lobes are not the only part of the cortex implicated in risky decision-making. The cingulate cortex, insula, temporo-parietal lobe as well as subcortical areas (e.g., ventral striatum), may respond to value according to the way it is assessed or objective features of choice alternatives (Clithero et al., 2009; Fitzgerald et al., 2010; Kahnt et al., 2010; Kahnt and Tobler, 2013). Age-related changes in the parietal cortex have been associated with age-dependent changes in risk perception (Grubb et al., 2016) and with the time-related processing of uncertain information (de Lange et al., 2010; Bode et al., 2012). All these areas (particularly the frontal lobes) have plentiful connections with subcortical structures such as the amygdala. Stress could therefore impair the process of decision-making by actions on this system, rather than on individual components of it (Maier et al., 2015).

The amygdala has been implicated in both cognitive and emotional components of risk-taking, another example of the blurred boundary between them (Bhatt et al., 2012). Since there is a profusion of androgen and glucocorticoid receptors in the amygdala, this is one avenue by which either hormone could influence financial risk-taking in a variety of ways, including the influence of testosterone on estimations of trustworthiness (in women; Bos et al., 2012). The amygdala is concerned with the regulation of loss aversion (Sokol-Hessner et al., 2013), and damage to it reduces this aversion though without impairing the ability to recognize changes in monetary value (De Martino et al., 2010). It can only be surmised that testosterone, which has a similar action, may operate though the amygdala and its connections with the orbital frontal cortex. Prediction of outcomes, and hence the risk associated with them, is also a function of this system (Dolan, 2007); there is as yet no clear evidence that either steroid alters the way this information is obtained or used, though since both alter risk appetite and, in the case of testosterone, estimates of expected outcome, it is highly likely that information processing is affected. Incidentally, although the hippocampus has high concentrations of glucocorticoid receptors (Gray et al., 2017), it has not, so far, been implicated in neural processes affecting risk appetite. Corticoids have a pronounced suppressive action on the formation of new neurons in the hippocampus (Cameron and Gould, 1994; Pinnock et al., 2007), though whether this influences financial decisions in the longer-term is also unknown.

## Financial Decisions as Conflict

As already mentioned, there are striking parallels between the modern situation in which acute and highly significant decisions have to be taken in a financial context (e.g., by day traders, who are mostly male) and an older biological one in which males are required to make equally rapid decisions in the context of personal competition (for mates) or collaborative conflict (war). In both, the outcome of a wrong decision may be either personal loss (finance: money; conflict: loss of assets, wounds, death), whereas success brings not only personal gain but social acclaim and heightened status, or gain of corporate assets (finance: profits for the company, conflict: territory, access to mates and other assets). In both situations, current information on which decisions are made or risks taken is likely to be complex, rapidly changing and incomplete. In both, experience and temperament will contribute to the behavioral response to a current acute and risk-laden situation. Whilst these considerations apply most obviously to rapid and possibly life-changing decisions in both contexts, more deliberate assessment of risks also occur in both conflicts and finance; for example, decisions on strategy, usually taken by older males (generals in war, managers in finance) than those who do the trading or the fighting. Much of the information on the factors that guide decisions made under the more primeval conditions of conflict will also apply to the more modern situations of finance. For example, testosterone reduces males’ tendency to reflect on decisions, a property which might be advantageous during fights as well as bond trading (Nave et al., 2017). It also increases confrontational decisions in a competitive financial encounter (Mehta et al., 2017). Though many studies focus on one or other steroid, it should be noted that both testosterone and cortisol do not act alone, but in the context of many factors, including interactions between the two hormones themselves. For example, the action of testosterone may depend on coincident levels or changes in cortisol, and vice versa (Mehta and Josephs, 2010; Mehta et al., 2015). Both testosterone and cortisol, the former implicated in the (male) involvement in competition, aggression and war, the latter in the stress response to urgent need and demand, and the areas of the brain on which they act, will thus play roles in finance that are foreshadowed by a more ancient biological imperative (Herbert, 2017).

Comparing physical conflicts with financial exchanges suggests another parallel: that a beneficial outcome for a group may not always be the same as for an individual, and this will affect not only processing of information, risk-assessment and decision-making, but also the individual endocrine response to a given situation. For example, in war it may be that the sacrifice of an individual works to the group’s advantage; in financial terms, risk-taking by an individual, though detrimental to that individual’s success, may provide information that benefits the group (company). This may be reflected in the function of both testosterone and cortisol. For example, since testosterone increases risk-appetite, it may be that there are situations in which this is related to in over-ambitious actions that result in individual loss, but future gain for the group. Group interactions, as well as personal characteristics, will therefore influence risk-taking. Similar ideas apply to cortisol. Excessive stress may impair individual performance, but provide corporate benefits in terms of heightened caution or inter-personal learning. It is thus difficult to define “optimal” levels of either hormone: this will depend both on the qualities of the individual and of the group of which he is a member (females will need a separate analysis). That is not to say that non-optimal levels of either cortisol or testosterone may not occur, and which contribute to disadvantageous outcomes both for the individual and the group. The lack of information on hormonal responses and correlations in real-life situations, and ignorance about the background on which they act (context, experience, personality, genetic variations etc.) means that we are currently unable to assess these factors with any certainty.

## Neuroscience vs. Economics

The focus of neuroscience is primarily on the role of hormones in the way that individuals respond to financial risks. This includes a wide range of related disciplines associated with decision-making, including psychological and social factors that influence such decisions. Neuroscience is thus concerned mostly with individual variation in risk assessment and decisions consequent on this neural process. Economists, on the other hand, are primarily interested in the way this affects the price of assets, and the occurrence of bubbles and crashes (i.e., market stability). Their concern is not so much with individuals and how they might vary, but with the results that corporate decisions might have on the market. The recent realization that there is considerable overlap between the two approaches has given rise to the relatively new topic of neuroeconomics (Camerer and Fehr, 2006; Camerer, 2008).

An example is the action of either testosterone or cortisol on financial decisions. This will have a median (average) effect on individuals, but modulated by genetic constitution, early and recent experience, and social context. Whereas individual behavior is unlikely to influence asset prices, large-scale median action may well do so. This may be one result of a general effect on, say, assessment of risk or optimism about outcome, but also on socially-determined responses such as “herding” (see above). It is important to distinguish simultaneous actions, prompted by equivalent information, from concerted actions that occur in the absence of new information or even despite it, driven by imitation or false (irrational) belief in private information held by others (herding). This may result in a cascade in which progressively more members of a particular financial community (e.g., a trading floor) follow each other (Bikhchandani and Sharma, 2001).

Collective decisions made independently, but influenced overall by either testosterone or cortisol (or both), may also have a de-stabilizing effect on markets. It is therefore relevant that those concerned with trading should pay attention to the effects these two steroids (as well as the numerous other factors) have on individual or collective responses to a given market situation. Alterations in biases, emotions, risk-assessment and cognitive appraisal (Kahneman, 2011), all influenced by hormones, can be powerful drivers of markets. But they will not necessarily be the same in everyone, as repeatedly emphasized in this article. So, in addition to knowledge about the overall effects of hormones, the financial world also needs to understand how these may be moderated individually. Despite the current interest in neuroeconomics, financiers would do well to take greater interest in the way that individual decisions are made, including the powerful effects of hormones and their actions on emotion and cognition, whereas neuroscience needs to understand better the impact on the financial world of risk-laden decisions taken under duress and the consequences these may have for an economy.

## Conclusion

Much of this review has been concerned with experimental or laboratory studies on the role of testosterone or cortisol in risky financial decisions. Though these have been, to an extent, informative, there is a great need for two further lines of enquiry: studies on the effects of either hormone in real-life situations, difficult but not impossible, and the contribution that individual genetic variations make to the effects that either hormone has in situations in which they may play a part, or to propensities for individuals to engage in risky financial behavior either as a profession or in everyday life. Although it is always possible to characterize the roles of hormones of the basis of mean or median effects, another aspect of equal interest is the extent to which the financial behavior of individuals varies in their response to their own hormones, and the ways this comes about.

## Author Contributions

The author confirms being the sole contributor of this work and approved it for publication.

## Conflict of Interest Statement

The author declares that the research was conducted in the absence of any commercial or financial relationships that could be construed as a potential conflict of interest.
